# Supplementary material for: Particle Separation in a Microchannel with a T-Shaped Cross-Section Using Co-Flow of Newtonian and Viscoelastic Fluids
Source: Micromachines (Basel). 2023 Sep 28;14(10):1863. doi: 10.3390/mi14101863 (PMC10608855; doi:10.3390/mi14101863)
Supplement: Supplementary file 1 [file micromachines-14-01863-s001.zip › micromachines-2611481-supplementary.pdf]

micromachines

Article

Particle separation in microchannel with T-shaped cross-section using co-flow of Newtonian and viscoelastic fluids

Jinhyeuk Song [1], Jaekyeong Jang [2], Taehoon Kim [1,2]* and Younghak Cho [1,2]*

[1] Department of Mechanical System Design Engineering, Seoul National University of Science & Technology, 232 Gongneung-ro, Nowon-gu, Seoul 01811, Republic of Korea; toptree2@naver.com (J.S.)
[2] Department of Mechanical Design and Robot Engineering, Seoul National University of Science & Technology, 232 Gongneung-ro, Nowon-gu, Seoul 01811, Republic of Korea; jjangy5720@naver.com (J.J.)
* Correspondence: tkim99@seoultech.ac.kr (T.K.); yhcho@seoultech.ac.kr (Y.C.); Tel.: +82-2-970-6340 (T.K.); +82-2-970-6361 (Y.C.)

Abstract: In this study, we investigated the particle separation phenomenon in a microchannel with a T-shaped cross-section, a unique design detailed in our previous study. Utilizing a co-flow system within this T-shaped microchannel, we examined two types of flow configuration: one where a Newtonian fluid served as the inner fluid and a viscoelastic fluid as the outer fluid (Newtonian/viscoelastic), and another where both the inner and outer fluids were Newtonian fluids (Newtonian/Newtonian). We introduced a mixture of three differently sized particles into the microchannel through the outer fluid, and observed that the co-flow of Newtonian/viscoelastic fluids effectively separated particles based on their size compared to Newtonian/Newtonian fluids. In this context, we evaluated and compared the particle separation efficiency, recovery rate, and enrichment factor across both co-flow configurations. The Newtonian/viscoelastic co-flow system demonstrated a superior efficiency and recovery ratio when compared to the Newtonian/Newtonian system. Additionally, we assessed the influence of the flow rate ratio between the inner and outer fluids on particle separation within each co-flow system. Our results indicated that increasing the flow rate ratio enhanced the separation efficiency, particularly in the Newtonian/viscoelastic co-flow configuration. Consequently, this study substantiates the potential of utilizing a Newtonian/viscoelastic co-flow system in a T-shaped straight microchannel for the simultaneous separation of three differently sized particles.

Keywords: T-shaped cross-section; particle separation; co-flow; Newtonian fluid; viscoelastic fluid

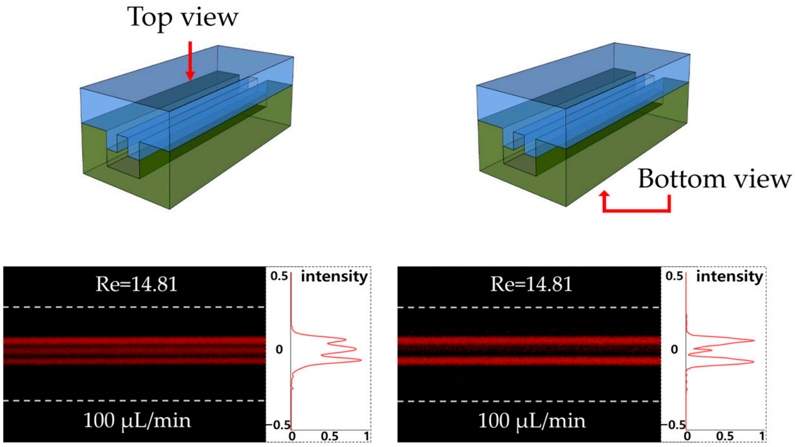

Figure S1. Fluorescent images and intensity graphs of particle (12 μm) focusing when viewed from the top and bottom under DI water

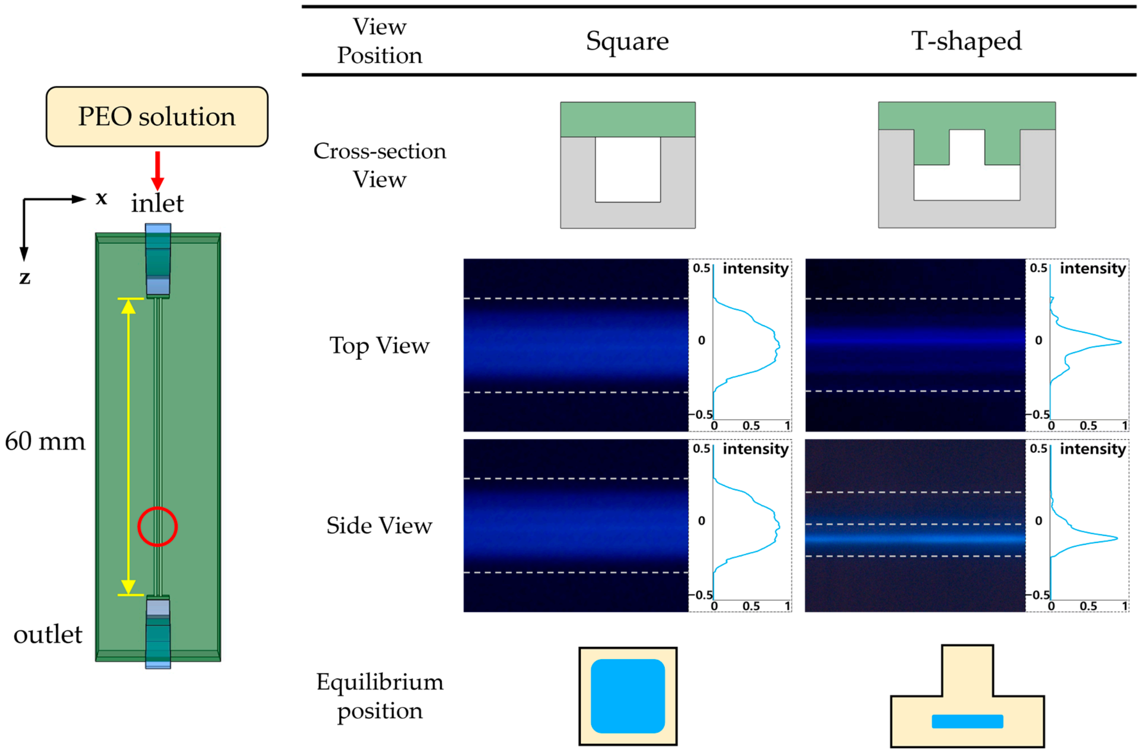

Figure S2. Comparison results in square channel and T-shaped cross-section channel with same hydraulic diameter under sheathless flow of PEO solution for 2.1 μm particles.

Table S1. Separation efficiency, recovery ratio and enrichment factor of 2.1 μm, 5.0 μm, and 12 μm particles. The total flow rate and flow rate ratio are 210 μL/min and 6.

	Factor	1 : 6		
		2.1 μm	5.0 μm	12 μm
Case 1	Separation efficiency(%)	95.08	94.01	95
	Recovery ratio(%)	93.25	92.05	93.44
	Enrichment factor	26.4	20.83	103.5
Case 2	Separation efficiency(%)	93.13	10.54	13.51
	Recovery ratio(%)	91.21	10.12	13.42
	Enrichment factor	1.26	0.79	33.28

Table S2. Separation efficiency of 2.1, 5.0, and 12 μm particles (average of three determinations ± standard deviation). The total flow rate and flow rate ratio are 210 μL/min and 6.

**Efficiency (%) ± SD (%) (n=3)**									
Case	1 : 6								
	(2.1, 5.0 μm)		(2.1, 12 μm)		(5.0, 12 μm)		(2.1, 5.0, 12 μm)		
	2.1 μm	5.0 μm	2.1 μm	12 μm	5.0 μm	12 μm	2.1 μm	5.0 μm	12 μm
Case 1	97.22 ± 0.2	95.13 ± 1.1	97.37 ± 0.1	98.22 ± 0.4	98.18 ± 0.3	95.03 ± 1.0	95.08 ± 0.3	94.01 ± 0.6	95.00 ± 1.2
Case 2	97.52 ± 0.9	9.64 ± 0.9	99.32 ± 0.1	16.08 ± 1.4	99.49 ± 0.1	13.79 ± 1.4	93.13 ± 0.9	10.54 ± 0.8	13.51 ± 1.4
